# TREM-1 Promotes Pancreatitis-Associated Intestinal Barrier Dysfunction

**DOI:** 10.1155/2012/720865

**Published:** 2012-05-07

**Authors:** Shengchun Dang, Yao Shen, Kai Yin, Jianxin Zhang

**Affiliations:** ^1^Department of General Surgery, Affiliated Hospital of Jiangsu University, Zhenjiang 212001, China; ^2^School of Clinical Medicine, Jiangsu University, Zhenjiang 212013, China

## Abstract

Severe acute pancreatitis (SAP) can cause intestinal barrier dysfunction (IBD), which significantly increases the disease severity and risk of mortality. We hypothesized that the innate immunity- and inflammatory-related protein-triggering receptor expressed on myeloid cells-1 (TREM-1) contributes to this complication of SAP. Thus, we investigated the effect of TREM-1 pathway modulation on a rat model of pancreatitis-associated IBD. In this study we sought to clarify the role of TREM-1 in the pathophysiology of intestinal barrier dysfunction in SAP. Specifically, we evaluated levels of serum TREM-1 and membrane-bound TREM-1 in the intestine and pancreas from an animal model of experimentally induced SAP. TREM-1 pathway blockade by LP17 treatment may suppress pancreatitis-associated IBD and ameliorate the damage to the intestinal mucosa barrier.

## 1. Introduction

Severe acute pancreatitis (SAP) can readily progress from a localized inflammation of the pancreas to a systemic disease and may lead to multiple organ dysfunction syndrome (MODS). The intestinal mucosal barrier plays a pivotal role in the pathophysiology of SAP, as the intestine is a primary target organ of inflammation-induced dysfunction and an initiator of MODS. A large number of studies have demonstrated that SAP is strongly associated with development of intestinal barrier dysfunction (IBD) [1, 2]. On the other hand, IBD caused by bacterial translocation, intestinal infection, or endotoxin exposure can aggravate SAP, and this complicating event has been proposed as a major cause of SAP death [3]. Unfortunately, the underlying molecular mechanisms that mediate the relationship between injury of the intestinal mucosa barrier and SAP remain to be fully elucidated.

The triggering receptor expressed on myeloid cells-1 (TREM-1) protein was recently described as a diagnostic marker of inflammation, as it is induced in the presence of bacteria or endotoxin. TREM-1 is mainly expressed on neutrophils and monocytes/macrophages where it functions in synergy with Toll-like receptor-mediated signals to increase the release of proinflammatory factors, such as TNF-*α* and IL-l*β* [4, 5]. Hence, TREM-1 acts as a critical amplifier of inflammatory signaling in response to lipopolysaccharide (LPS) and other microbial products [6].

Patients with acute pancreatitis (AP) are characterized by a high expression level of TREM-1 mRNA in leukocytes [7]. Moreover, intestinal expression of TREM-1 is upregulated in IBDs and correlates with disease severity [8]. Recent investigations have demonstrated that membrane-bound TREM-1 protein is increased in the pancreas, liver, and kidney of patients with SAP, suggesting that TREM-1 may act as an important mediator of inflammation and subsequent extrapancreatic organ injury in SAP [9].

In this study we sought to clarify the role of TREM-1 in the pathophysiology of IBD in SAP. Specifically, we evaluated levels of serum TREM-1 and membrane-bound TREM-1 in the intestine and pancreas from an animal model of experimentally induced SAP. Based on the TREM-1 sequence in the GenBank/EMBL/DDBJ (under accession nos. AF287008 and AF241219); a highly conserved domain (LQVTDSGLYRCVIYHPP) in rats, mice, and humans in the extracellular region of the protein was chemically synthesized as a COOH terminally amidated peptide (Pepscan Systems) and was designated as LP17 [10, 11]. LP17 can compete with the natural ligand of TREM-1 for binding and is believed to act as a so-called decoy receptor. The correct peptide was obtained in >99% yield and was homogeneous after preparative purification, as confirmed by mass spectrometry and analytic reversed-phase high-performance liquid chromatography. A scrambled peptide containing the same amino acids as LP17, but in a completely different sequence order (TDSRCVIGLYHPPLQVY), was similarly synthesized and designated as TY17 to serve as a negative control. Both in vitro and in vivo treatment with LP17, a synthetic peptide blocker of the TREM-1 pathway, efficiently suppressed and prevented the detrimental effects of proinflammatory cytokines. Our findings indicated that blockade of the TREM-1 pathway by LP17 mitigates the inflammatory response associated with SAP and provides therapeutic protection from intestinal mucosal damage. 

## 2. Materials and Methods

### 2.1. Materials

#### 2.1.1. Animals

Male Wistar rats (weighing 250–300 g, 9 weeks old) were purchased from the Laboratory Animal Center of Jiangsu University. The rats were allowed to acclimatize to the laboratory conditions for 7 d under a 12 h light-dark cycle at a constant temperature of 21 ± 1°C. The animals were fasted for 12 h before experiments but allowed free access to water. All experimental procedures were performed in accordance with the Guide for the Care and Use of Laboratory Animals and approved by the Animal Ethics Committee of Jiangsu University, China.

### 2.2. Induction of Experimental SAP

After 12 h of fasting, animals were anesthetized by intraperitoneal injection of 50 mg/kg phenobarbital, and a midline laparotomy was performed. SAP was induced by retrograde injection of 5% sodium taurocholate (TCA; 1 mL/kg body weight) into the biliopancreatic duct in a pressure- and volume-controlled manner over 10 min. Sham-operated rats were used as non-SAP (healthy) controls, in which only the laparotomy was performed.

### 2.3. Experimental Grouping

A total of 64 healthy male Wistar rats were randomly divided into sham-operated group, SAP group, SAP + LP17, and SAP + TY17 group with 16 rats in each group. Eight rats in each group were only used for intestinal permeability assessment. Based on the results of previous studies, the rat intestinal injury has been found serious at 6 h after TCA injection; therefore, rats were used 6 h after injection to investigate SAP associated with IBD [12]. LP17 or TY17 was administered intravenously (1.0 mg in 0.1 mL of saline) just before the induction of pancreatitis.

### 2.4. Rat Blood Samples

Six hours after the rat SAP model was established, the rats were re-anesthetized, and blood samples were drawn from the heart. The blood samples were individually collected into dry, sterile test tubes and centrifuged at 2500× g for 15 min; serum was removed and stored at −80°C until use.

### 2.5. Analysis of Serum TREM-1, Serum Diamine Oxidase (DAO), and D-Lactate

The amount of TREM-1 in rat blood serum was determined in duplicate using ELISA kits (GBD, Canada) as described by the manufacturer's protocol. The serums were quantitated for DAO and D-lactate concentrations using commercial kits and following the manufacturer's protocol (Genmed, China) by spectrophotometric measurements.

### 2.6. Analysis of TREM-1, TNF-*α*, and IL-1*β* mRNA in Intestine and Pancreas by Real-Time Quantitative (qRT-)PCR

Distal ileums and pancreatic tissue samples were collected and stored at −80°C when the animals were sacrificed. Total RNA was isolated from rat intestinal mucosa and pancreas using TRIzol reagent (Invitrogen Corp., USA), according to the manufacturer's instructions. Complementary DNA (cDNA) was synthesized using a reverse-transcription kit (Toyoba, Japan). The cDNAs were subjected to qRT-PCR using the SYBR Premix Ex Taq (Takara, Japan) on a Rotor-Gene 6000 instrument. Primers were designed as follows: rat TREM-1, forward 5′-AAGTATGCCAGAAGCAGGAAGG-3′ and reverse 5′-GGTAGGGTCATCTTTCAGGGTGT-3′; rat TNF-*α*, forward 5′-CGTCGTAGCAAACCACCAAG-3′ and reverse 5′-CACAGAGCAATGACTCCAAAG-3′; rat IL-1*β*, forward 5′-AATACCACTTGTTGGCTTA-3′ and reverse 5′-TGTGATGTTCCCATTAGAC-3′; rat *β*-actin, forward 5′-CCTGTACGCCAACACAGTGC-3′ and reverse 5′-ATACTCCTGCTTGCTGATCC-3′. The comparative Ct method (2^−ΔΔ^Ct) was used to calculate the relative gene expression levels [13].

### 2.7. Intestinal Permeability Assessment

Intestinal permeability was quantified using the lactulose/mannitol (L/M) test as previously described by Hang et al. [14] with high-pressure liquid chromatograph (HPLC). Eight hours before the animals were sacrificed at 6 h after TCA injection in each group, the rats with their bladders emptied were given the test solution of 2 mL by gastric tube feeding, containing 100 mg lactulose and 60 mg mannitol. All urine was collected for 8 hours through the metabolic cage and stored at −20°C for further analysis. Results were expressed as a ratio of the two molecules excreted.

### 2.8. Testing for Bacterial Translocation

Mesenteric lymph nodes in 8 rats in each group were taken for bacteriologic cultures. Collected tissue samples were weighed, and 0.5 g of each was homogenized in a tissue grinder with 4.5 mL sterile saline solutions (SS) to made 10% tissue-slurry, then 0.1 mL solutions immediately cultured in agar-blood medium plates. Inoculated plates were incubated at 37°C for 24 hours. Cultures were considered positive when more than 100 colonies per gram of tissue were found.

### 2.9. Pathological Examination

Upon excision, the pancreas and distal ilea (5 cm long) were promptly fixed in 4% formalin and embedded in paraffin wax, following standard procedures. Paraffin-embedded pancreas and distal ilea were sectioned (5 *μ*m), then stained with hematoxylin and eosin (H&E). The degree of mucosal damage was graded according to the standard scale of Chiu et al. [15]: 0 = normal mucosa, 1 = development of subepithelial space at the tip of the villus, 2 = extension of the space with epithelial lifting, 3 = massive epithelial lifting, 4 = denuded villi, and 5 = disintegration of the lamina propria.

### 2.10. Statistical Analysis

All the data were expressed as mean ± standard deviations (SDs) using SPSS statistical software (version 18.0) and GraphPad Prism 5.0. If equal variances were assumed, one-way ANOVA was used. The degree of mucosal damage in each group was evaluated using the Mann-Whitney *U* test. *P* < 0.05 was considered statistically significant.

## 3. Results

### 3.1. Levels of TREM-1 in Serum

**Figure 1:**
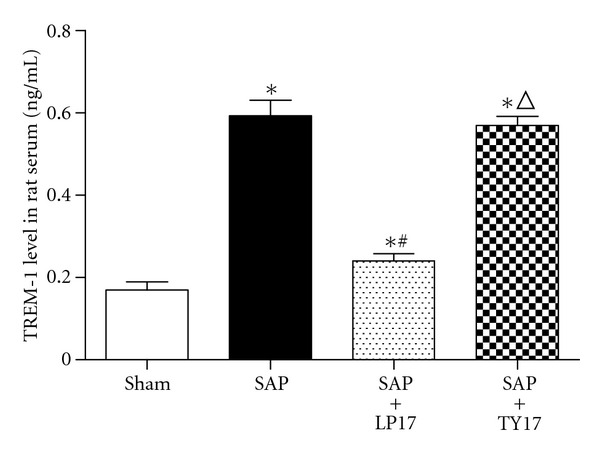
Levels of TREM-1 in serum in each group. *P versus sham group, ^#^ *P* versus SAP group, ^Δ^ *P* versus SAP + LP17 group (*P* < 0.05, as below).

Levels of soluble TREM-1 in serum in the SAP group and SAP + LP17 group were significantly higher than those in the sham group and SAP + LP17 group (*P* < 0.05, Figure 1).

### 3.2. The Expression of TREM-1, TNF-*α*, and IL-1*β* mRNA

**Figure 2:**
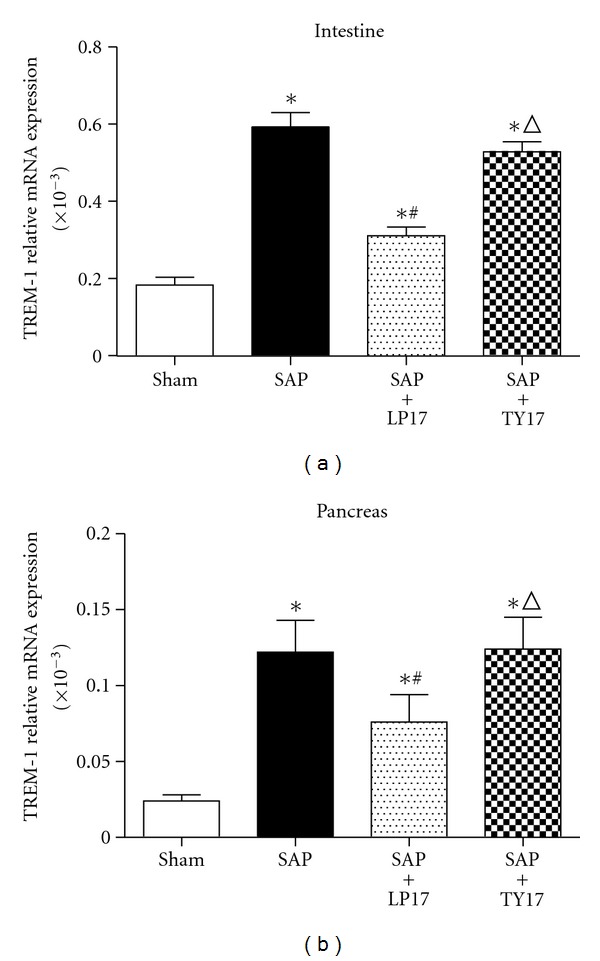
The expression of TREM-1 in the intestinal and pancreatic tissues.

**Figure 3:**
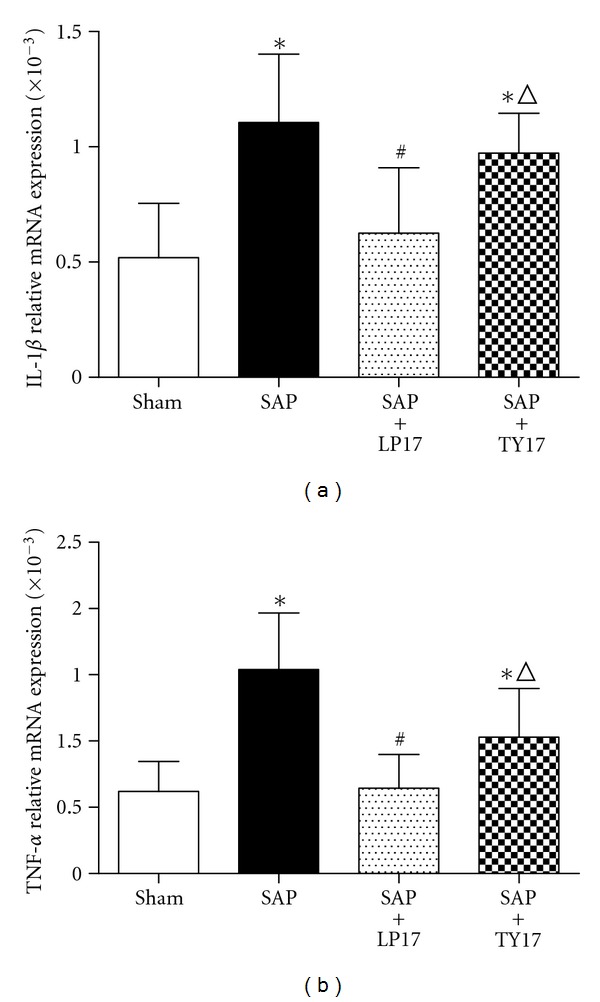
The expression of IL-1*β* (a) and TNF-*α* (b) mRNA in the intestinal tissues.

Our results indicated that the TREM-1, TNF-*α*, and IL-1*β* mRNA expressed in the SAP group and SAP + LP17 group were significantly higher than those in the sham group and SAP + LP17 group in intestinal tissues (*P* < 0.05). Pretreatment with LP17 significantly decreased the expression of TREM-1, TNF-*α*, and IL-1*β* levels in the SAP group. The TREM-1 expressed in pancreatic tissues in the SAP group and SAP + LP17 group was significantly higher than that in the sham group and SAP + LP17 group. These data suggested that LP17 may attenuate intestinal mucosa injury induced by the inflammatory response mediated by these two cytokines (Figures 2 and 3).

### 3.3. Levels of DAO and D-Lactate in Serum

**Figure 4:**
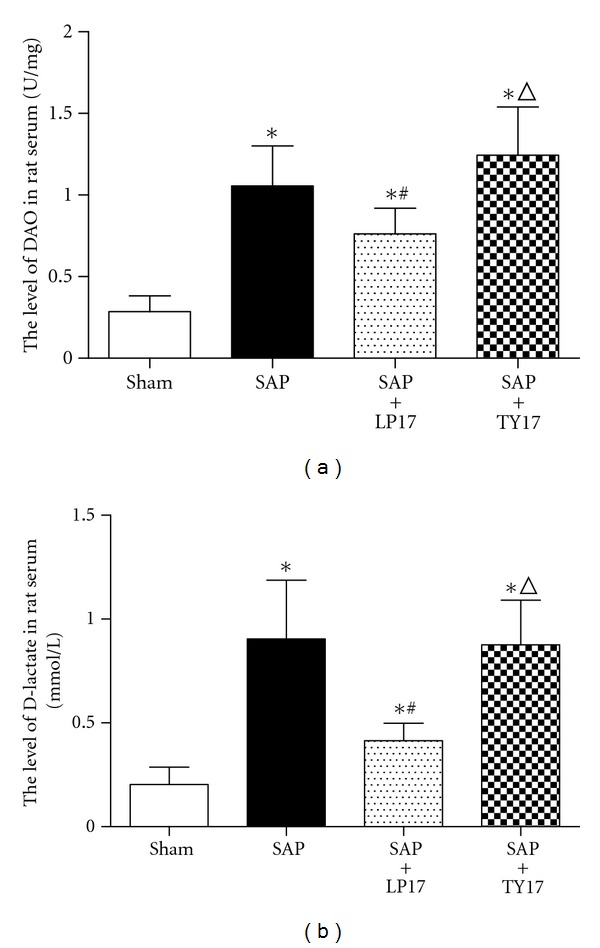
Levels of DAO and D-lactate in serum.

Spectrophotometry results revealed that the levels of DAO and D-lactate were distinctly higher in the SAP group and SAP + LP17 group than those in the sham group. However, the levels of DAO and D-lactate in the SAP + LP17 group were lower than either the SAP group or the SAP + TY17 group (*P* < 0.05, Figure 4).

### 3.4. Intestinal Permeability (Lactulose and Mannitol Assay)

**Figure 5:**
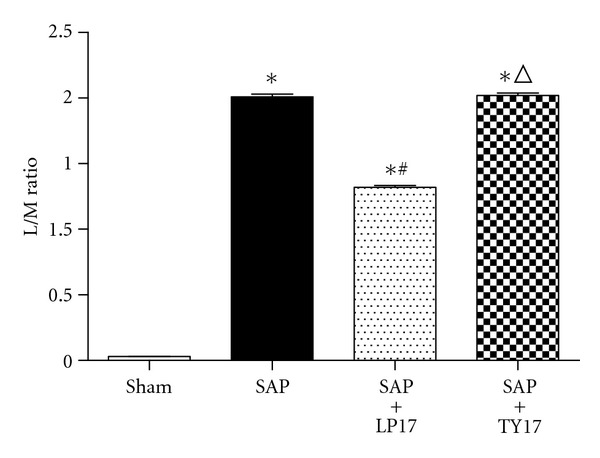
Intestinal permeability, as assessed by the lactulose/mannitol (L/M) excretion ratio.

As shown in Figure 5, intestinal permeabilities in the SAP group and the SAP + LP17 group were significantly higher than those in the sham group and SAP + LP17 group. LP17 administration to SAP caused a clear deterioration in the mucosal permeability, as assessed by the L/M ratio than that in the SAP group and the SAP + TY17 group (*P* < 0.05).

### 3.5. Quantitative Bacteriologic Culture of MLNs

**Figure 6:**
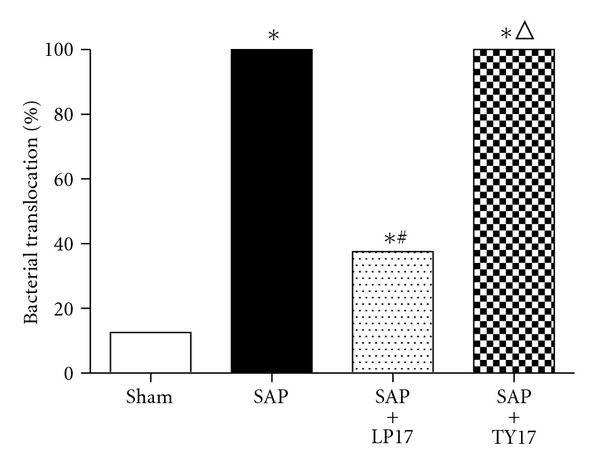
Quantitative bacteriologic culture of MLNs.

Tissue cultures tested positive for bacteria in all animals in the SAP group and SAP + LP17 group. In contrast, only one of seven animals in the sham group showed positive tissue cultures. The percentages of positive harvested MLNs in the SAP + LP17 group were lower than either the SAP group or the SAP + TY17 group. Data are presented as percentages of harvested tissue samples (*P* < 0.05, Figure 6).

### 3.6. Pathologic Examination of Pancreas and Intestinal Mucosa

**Gross Observation**

In the sham group, the pancreas showed no significant changes. In the SAP group or the SAP + TY17 group, bloody ascites in the abdominal cavity as well as pancreatic congestion, edema, hemorrhage, and necrosis, was noted. In the SAP + LP17 group; pathological changes were milder than those in the SAP group or the SAP + TY17 group.

**Light Microscopy**

**Figure 7:**
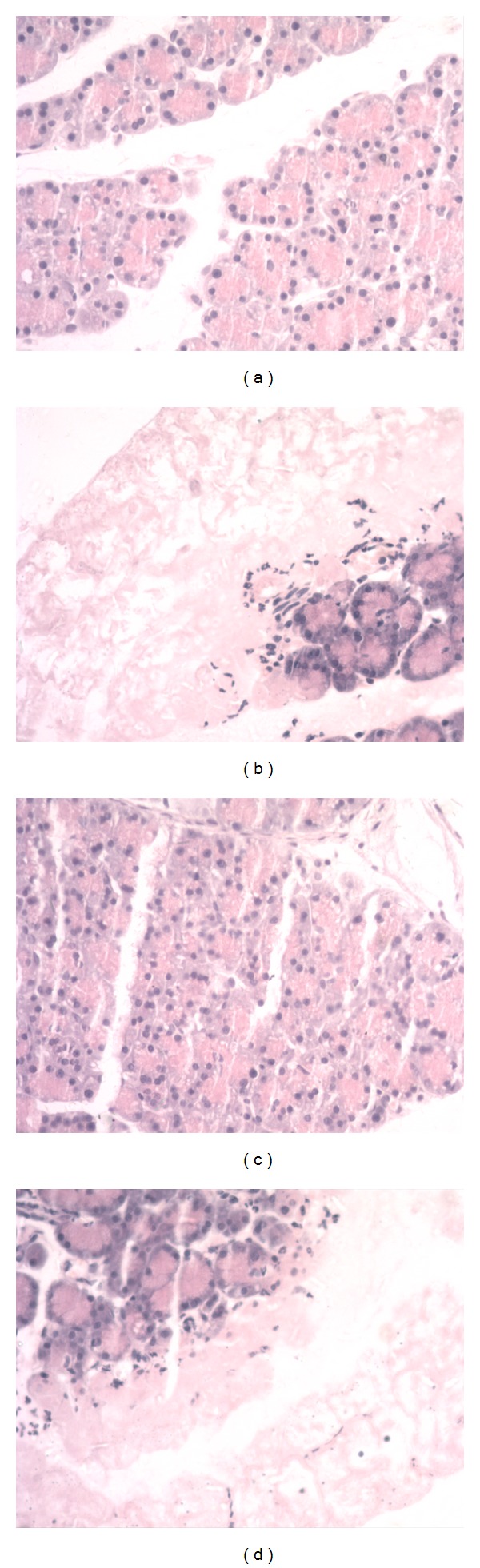
Pathological changes in the pancreas. (a) Sham, (b) SAP, (c) SAP + LP17, (d) SAP + TY17.

In the sham group, the pancreas of rats showed no morphological or structural abnormalities Figure 7(a); in SAP group or the SAP + TY17 group there were varying degrees of focal interlobular edema, necrotic areas without structure, and red blood cells in the tissue space, as well as massive inflammatory cell infiltration Figures 7(b) and 7(d); in the SAP + LP17 group Figure 7(c), pancreatic edema, hemorrhage, and necrosis, as well as inflammatory cell infiltration, were milder than in SAP group or the SAP + TY17 group (Figure 7).

**Figure 8:**
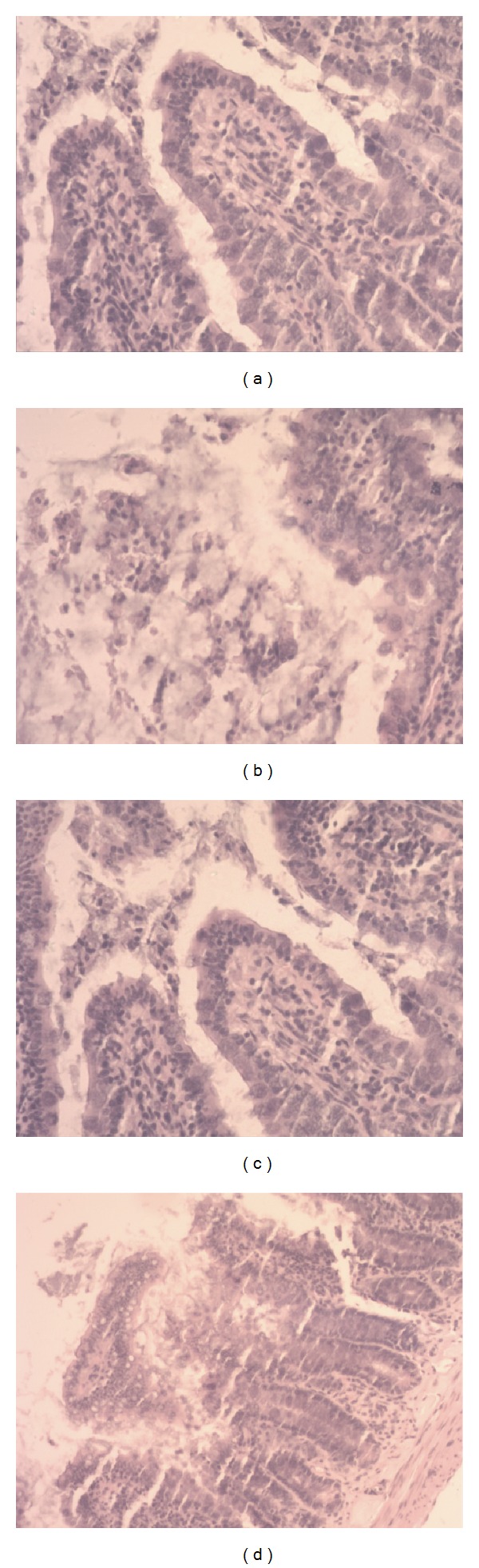
Histological findings of the intestinal mucosa. (a) Sham, (b) SAP, (c) SAP + LP17, (d) SAP + TY17.

**Figure 9:**
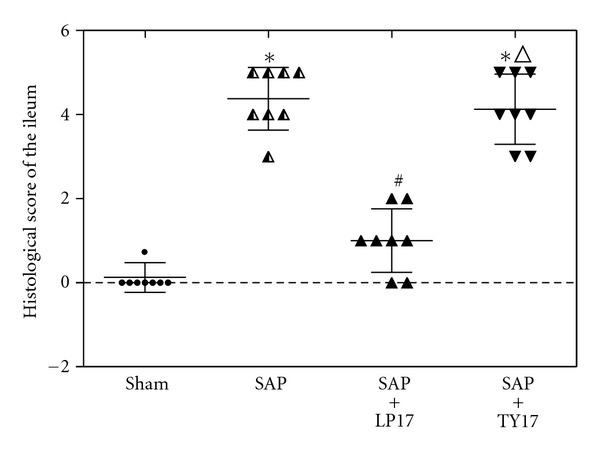
Histological score of the ileum at 6 h after the induction of SAP.

After induction of SAP, the rats exhibited characteristic pathological changes such as broadened villi, infiltration of inflammatory cells, and parenchyma hemorrhage in the SAP group or the SAP + TY17 group. Treatment with LP17 ameliorated these effects, and only mild villous edema and decreased red blood cells in the interstitial space were observed (Figure 8). The degree of intestinal pathological injury is shown in (Figure 9).

## 4. Discussion

The results of our study show that increases in serum and membrane-bound TREM-1 levels are significantly associated with SAP disease severity and IBD in rats. Moreover, we also found that blockade of the TREM-1 pathway, by means of LP17 treatment, can ameliorate intestinal mucosal damage and systemic inflammation, and this finding suggests that TREM-1 may be a potential therapeutic target for SAP-associated IBD.

 The intestine represents the largest immune organ of the human body. The immune function is an important part of the intestinal epithelial barrier, which actively prevents and resolves pathogenic infection. Intestinal mucosa injury in case of SAP is one of the main reasons for the accelerated aggravation of this disease. Unfortunately, the underlying molecular mechanisms involved have yet to be completely understood, and an effective means to prevent SAP complications related to IBD has yet to developed.

 It was initially reported that TREM-1 acted as an amplifier of inflammation by enhancing degranulation and secretion of proinflammatory mediators. Increased levels of soluble TREM-1 (sTREM-1) have been detected in patients and animals suffering from a variety of infectious and noninfectious diseases, often correlating with disease severity [16–18]. In patients suffering from AP, serum sTREM-1 was found to be higher than that in healthy individuals [19]. In our study using the rat model of induced SAP, both serum soluble TREM-1 levels and membrane-bound TREM-1 expression in intestine tissue were significantly increased at 6 h after the induction of pancreatitis, and those increased levels persisted over time. Yasuda et al. reported that the early phase and the late phase (18 h after the induction) of AP were associated with blood endotoxin and bacterial translocation events [20]. Another recent study found that Gram-negative bacteria-derived bacterial DNA was present at detectable levels in AP patients' serum [21]. Therefore, we theorized that serum sTREM-1 levels and membrane-bound TREM-1 expression in the very early phase of SAP would be upregulated due to the nonmicrobial inflammation response (or the systemic inflammatory response) [22, 23]. Subsequently, in the late phase of SAP, sTREM-1 in serum may be increased in response to undetectable microbes or endotoxin. The results of this study showed that the expression levels of TREM-1 protein and the pathological severity scores of the intestinal mucosa of SAP rats in SAP group were significantly higher than those in the sham group, indicating that the mucosa damage may be related to upregulation of TREM-1 protein. Pancreatitis-associated IBD progresses as blood monocytes are recruited and produce proinflammatory cytokines; hence, we think membrane-bound TREM-1 upregulation in the intestine and pancreas is increased on the infiltrating neutrophils in response to the inflammation cascade in process, and these systemic responses may be closely related to organ damage (and MODS).

To therapeutically block the TREM-1 pathway, we administered the peptide (LP17) by intravenous injection. This peptide had been originally designed by Gibot et al. and was derived from the extracellular domain of TREM-1. The LP17 has been demonstrated by others to be of therapeutic benefit during sepsis and acute mesenteric ischemia-reperfusion [24, 25]. In addition, another recent study demonstrated that TREM-1 expression was associated with the common IBDs, ulcerative colitis (UC), and Crohn's disease (CD). In experimental mouse models of UC and in human patients with CD, TREM-1 expression in the intestine was upregulated and correlated with disease severity. Furthermore, blocking TREM-1 by means of treatment with LP17 was shown to decrease disease severity. In our study, we constructed the SAP animal model by treatment with TCA and detected the two characteristic markers of intestinal barrier dysfunction caused by SAP, D-lactate, and DAO. We found that LP17 can reduce the levels of D-lactate and DAO in TCA-induced SAP rats. This finding indicated that treatment with LP17 significantly improved intestinal barrier function and reduced the extent of intestinal mucosal damage associated with SAP.

The action of proinflammatory cytokines is another important injury-inducing mechanism that impacts the integrity of the intestinal mucosa. Excessive release of inflammatory mediators, such as IL-1*β* and TNF-*α*, can directly damage the intestinal mucosa barrier [26]. TNF-*α*, in particular, participates in the early steps of SAP and can induce expression of other inflammatory cytokines [27–29]. In addition, TNF-*α* is known to stimulate the generation of several injuring substances, such as nitric oxide (NO) and oxygen-free radicals, which can lead to further inflammatory cascade and amplification effects [30, 31]. TNF-*α* can also promote the permeability of intestinal mucosa barriers and break down the tight junctions so that intestinal bacteria may easily transverse into the mesenteric lymph nodes and other organs [32]. Continuous invasion of intestinal bacteria and endotoxin into the body will lead to the life-threatening systemic inflammatory response syndrome (known as SIRS) and MODS. IL-1*β* is another classic proinflammatory cytokine; it can destroy the endothelial barrier integrity by stimulating the release of inflammatory mediators from leukocytes and endothelial cells. On the other hand, compromised endothelial cells can enhance the synthesis and secretion of IL-1*β* [33]. Some results found that resident intestinal macrophages have an inherent ability to produce TNF-*α*. Activation of intestinal macrophages by unknown substances may contribute to the induction of TNF-*α* production, which causes the intestinal inflammation [34]. So we use LP17 to block the TREM-1 pathway of local intestinal macrophages and infiltrating neutrophils in intestine and observe the changes of TNF-*α* and IL-1*β* production.

To study the effect of LP17 on both of these proinflammatory cytokines, we used qRT-PCR to determine the mRNA level of IL-1*β* and TNF-*α* in intestinal mucosa. Our results showed that the expressions of IL-1*β* mRNA and TNF-*α* mRNA were significantly suppressed by LP17 treatment in our rat SAP models, suggesting that LP17 may exert protective effects on the small intestine by mitigating the inflammatory response. This result is in agreement with other related recent investigations. So we believe that LP17 can inhibit the expression of TREM-1 in both local intestinal macrophages and infiltrating neutrophils from blood.

In conclusion, our data suggest that TREM-1 participates in pancreatitis-associated IBD, and pretreatment with LP17 is able to reduce the severity of intestinal injury. LP17 acts to diminish the release of IL-1*β* and TNF-*α* in the intestine during the early phase of SAP. The modulation of TREM-1 signaling by the use of a synthetic peptide such as LP17 appears to be a promising therapeutic approach for the treatment of acute pancreatitis complicated by injury of the intestinal mucosa barrier.

Although LP17 treatment in our rat model was efficacious, some considerations should be noted. To obtain a successful model of SAP, we chose not to administer antibiotics to the rats, but in clinic most of the patients who have SAP receive early and adequate antibiotic therapy. The TREM-1 ligands are not known at present; future studies directed at identification of natural ligands of TREM-1 will advance our understanding of its physiological significance during an immune response against microbial and nonmicrobial inflammation.
